# INTRODUCING A NEW AGE-BASED DATASET FOR EVALUATING OCCUPATIONAL CHARACTERISTICS ACROSS THE LIFE COURSE

**DOI:** 10.1093/geroni/igad104.0438

**Published:** 2023-12-21

**Authors:** Qize Chen, Amanda Sonnega, Dawn Carr, Rebekah Carpenter, Katy (Qiuchang) Cao

**Affiliations:** University of Michigan, Ann Arbor, Michigan, United States; University of Michigan, Ann Arbor, Michigan, United States; Florida State University, Tallahassee, Florida, United States; Florida State University, Tallahassee, Florida, United States; Florida State University, Tallahassee, Florida, United States

## Abstract

The connection between early life career choices and later life outcomes is a topic of interest to gerontology researchers, but research has been limited by data availability. In this paper, we present a newly constructed LHMS-HRS-O*NET linked dataset. The 2017 Health and Retirement Study (HRS) Life History Mail Survey (LHMS) includes information on up to 10 jobs that respondents worked for more than a year. We consolidated this employment history with the biennial HRS Core survey data. Then, respondents’ occupations from the LHMS were mapped to the O*NET 26.1 Database to add information on their exposure to different work contexts, activities, and etc. The final dataset is a panel at individual-year level with annual occupational exposure from the first job after full-time education until the last job people held before 2017. The data covers over 4,000 individuals in the HRS with all O*NET measures available in O*NET 26.1. This presentation will demonstrate how researchers can use these data to calculate the cumulative occupational exposures for any period of one’s career and connect that information to socioeconomic, health, and other outcomes at older ages that are available in HRS. This new data is ideal for studying how full life histories of work are related to differential aging outcomes.

